# The Effect of Educational Intervention on Improvement of Breastfeeding Self-Efficacy: A Systematic Review and Meta-Analysis

**DOI:** 10.1155/2021/5522229

**Published:** 2021-08-10

**Authors:** Azam Maleki, Elham Faghihzadeh, Samaneh Youseflu

**Affiliations:** ^1^Social Determinants of Health Research Center, Zanjan University of Medical Sciences, Zanjan, Iran; ^2^Department of Epidemiology and Biostatistics, School of Medicine, Zanjan University of Medical Sciences, Zanjan, Iran; ^3^Department of Midwifery and Reproductive Health, Isfahan University of Medical Sciences, Isfahan, Iran

## Abstract

**Background:**

Self-efficacy is an important psychological and motivational factor in breastfeeding, and it is a valuable framework that predicts breastfeeding outcomes and demonstrates maternal confidence in breastfeeding. The meta-analysis evaluated the effectiveness of educational interventions on improving breastfeeding self-efficacy (BSE).

**Methods:**

The English and Persian databases including Medline, Embase, Cochrane Database of Systematic Reviews (CDSR), PubMed, Web of Science, Scopus, CINAHL, Sid, IRANDOC, and Marg-Iran were systematically searched for studies published from January 2005 to December 2020. The quality of studies was evaluated using the Cochrane risk of bias tool and the heterogeneity by *I*^2^ statistic. The extracted data were analyzed using RevMan 5 statistical software and presented using random effects standardized mean difference (SMD). The funnel plot was used for evaluating publication bias.

**Results:**

Results from 40 RCTs showed that educational intervention had a positive effect on the BSE compared with the usual/standard care (pooled SMD = 1.20; 95% CI = 0.75–1.64, *p* value <0.001). The subgroup analysis indicated that the educational intervention was based on theory, group class format, direct method education, during the first week of postpartum, doing during pregnancy, on primiparous women, and health center setting, and the Asian region has a more effect on BSE than the others.

**Conclusion:**

Breastfeeding education is considered an influential factor in the improvement of BSE. It is recommended that breastfeeding education should be continued for several weeks after childbirth for gaining its benefit. The Asian region has a more effect on BSE than the others. Therefore, it is important to add the values in content of education in each country.

## 1. Introduction

Breastfeeding has the health benefit and promotes physical and mental health of the mothers and their infants. So that infants who are exclusively breastfed for six months experience less morbidity than those who are partially breastfed [1]. Therefore, breastfeeding as a unique method of feeding and growth of infants in any situation and region of the world is recommended exclusively for the first 6 months of life [2]. Despite global strategy targets in low- and middle-income countries, only 37% of children younger than 6 months of age are exclusively breastfed that is below the WHO recommended rate of 50% [1]. It is even less in developed countries. Therefore, declining breastfeeding rates are universal concern [1, 2].

Self-efficacy is an important psychological and motivational factor in breastfeeding [3] and it is a valuable framework that predicts breastfeeding duration and demonstrates maternal confidence of breastfeeding [4, 5]. Consistent with Bandura's cognitive-social theory, self-efficacy as a cognitive dynamics process evaluates people's beliefs and their ability to perform healthy behaviors and contributes to their preventive behavior [6]. Dennis maintains that four important sources including performance accomplishment, vicarious experience, verbal persuasion, and physiologic responses can impress women's levels of BSE [4]. Each of them may affect the mothers perceive about her breastfeeding, and it informs her BSE [4].

Developing and applying effective educational programs for improving breastfeeding self-efficacy (BSE) are important concerns for health professionals, and it can help mothers initiate and maintain breastfeeding for six months after birth [7, 8]. In particular, breastfeeding educational programs have shown a positive effect on maternal breastfeeding behavior, awareness and attitude toward breastfeeding, continuity of breastfeeding, and BSE of mothers at six months after childbirth recently [5, 9, 10]. However, other studies have been reported conflicting results [11, 12]. In some studies, the effectiveness of educational programs on BSE has been reviewed. However, in these studies, the limit number of English language studies has been reviewed or the quality of the studies was not determined or type of study was included observational studies, or only one dimension of education, such as theory or telephone based, was considered [5, 13–15]. There is a wide variety in terms of the type and time of educational intervention. Therefore, reviewing the difference in setting, time, and type of educational intervention on breastfeeding self-efficacy is one of the important steps to provide practical support for developing effective educational programs and policy making. The aim of this systematic review and meta-analysis was to determine the effect of educational programs on BSE.

## 2. Materials and Methods

### 2.1. Search Strategy and Data Sources

**Figure 1:**
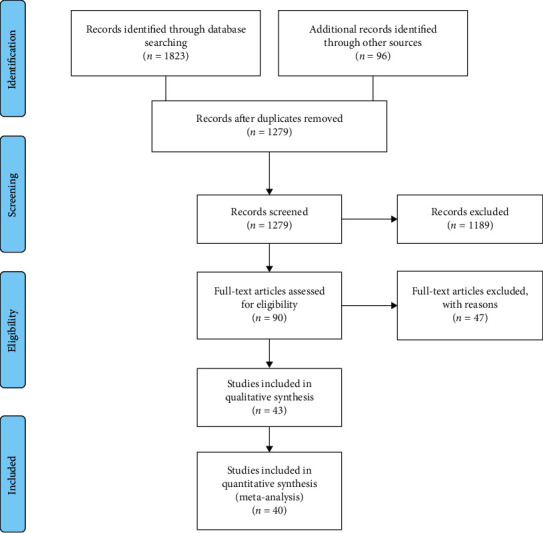
PRISMA flow diagram.

The English and Persian electronic databases including Medline, Embase, Cochrane Database of Systematic Reviews (CDSR), PubMed, Web of Science, Scopus, CINAHL, Sid, IRANDOC, and Mag-Iran were systematically searched for studies published from 2005 to December 2020 using the following search strategies in accordance with the Mesh browser keywords and free-text words: (Feeding*∗*OR Breast*∗*OR Breastfeeding OR “Breast Milk” OR “Human Milk” OR “Breast Milk” OR Lactation OR “Milk Secretion” OR Colostrum OR “Exclusive Breastfeeding”) AND (Pregnancy OR Gestation OR “Pregnant Woman”) AND (Postpartum OR Puerperium) AND (“Self-Efficacy” OR “Self-confidence” OR “Self-concept”) AND (Education*∗* OR “Health Education” OR Instruction*∗*OR Training) AND (“randomized controlled trial” OR “controlled clinical trial” OR randomized*∗* OR randomly*∗* OR trial*∗* OR groups). Moreover, reference lists of the identified articles were screened. The PRISMA checklist (Figure 1) was used for reporting the search result [16]. We used a search strategy, which has been developed in Medline and adapted for other databases. We also used manual approaches, in particular, hand-searching and perusing reference lists of articles to find additional studies for systematic review. Various grey literature databases (such as Open Grey SIGLE, NTIS, Global Index Medicus (GIM), Google Scholar, World Cat, UW Libraries Search), theses, and dissertations, as well as conference proceedings, were assessed for collecting unpublished data. This study was not registered in the PROSPRO database. This meta-analysis was performed and reported in adherence with Preferred Reporting Items for Systematic Reviews and Meta-Analysis.

### 2.2. Inclusion Criteria

The studies with the following criteria were included: (1) healthy mothers with healthy baby, (2) randomized controlled trials (RCTs) with at least two groups (control and intervention) that aimed to measure mother's BSE, (3) various forms of breastfeeding education (for example, didactic teaching session, face to face, indirect, individual, group, peer support, and workshop) combined with or without other interventions, (4) comparison groups were assigned to usual care and standard care, and (5) study published from Juan 2005 to December 2020 with restricted English and Persian. Due to the fact that the review of interventions in the last 15 years is critical for developing breastfeeding promotion strategy, the period for searching for articles in this review is limited to 2005–2020 years.

### 2.3. Exclusion Criteria

The studies with the following criteria were excluded: (1) the mothers with chronic/systemic disease, parents with preterm baby with/without admitted at NICU ward, and complication in the breast, such as mastitis, (2) a quasi-experimental study, meta-analysis, and cross-sectional and observational studies, (3) noneducational intervention such as kangaroo mother care, motivational interview, psychoeducational counseling approach, and relaxation tone, and (4) randomized controlled trials (RCTs) that measured the mothers' knowledge or practice of breastfeeding, or breastfeeding initiation or duration.

### 2.4. Outcome

In this study, the main outcome was breastfeeding self-efficacy. The subgroup analysis was done based on the type of education (telephone, theory based, and group/individual), time of education (pregnancy/postpartum), participants (primiparous/multiparous), follow-up period, region, study quality, and setting (hospital/health centers).

### 2.5. Data Extraction

The titles and abstracts of the eligible studies were independently extracted and screened by two authors (AM and SY), and duplicates were also removed. After providing the full texts of the studies, data extraction was done using a structured form included the name of the author, year of publication, location of study, type of intervention, the time of education, setting, participants, lengths and frequencies of sessions, the comparison group, sample size, measurement instrument, and results. Disagreements of the extractors were resolved by discussion or consultation with the third person.

### 2.6. Quality Assessment

**Figure 2:**
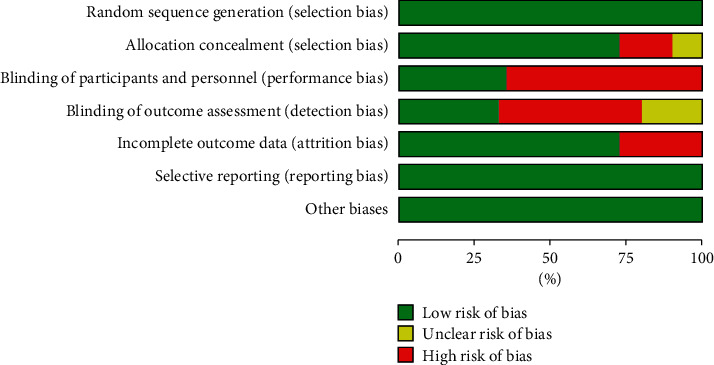
The results of Cochrane risk of bias tool for the evaluation of clinical trial quality.

Quality and risk of bias appraisal was conducted based on the guidelines of Cochrane Collaboration's tool for assessing the risk of bias in randomized trials [17]. 31 studies were considered having low risk of bias (Figure 2).

### 2.7. Analysis

**Figure 3:**
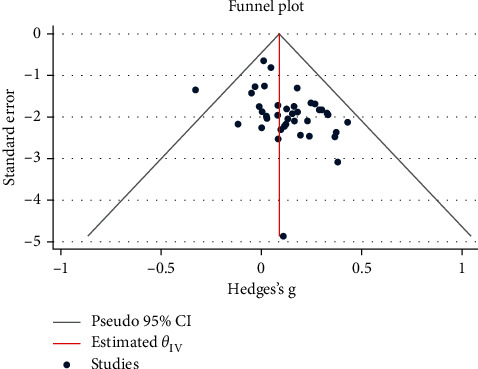
Inverted symmetrical funnel plot for showing publication bias.

RevMan Software Version 5 was used for analyses. Mean differences were used to find the effect for quantitative data. The heterogeneity of the studies was assessed using *I*-squared. Due to the high heterogeneity (*I*^2^ > 50%), the random effect was used instead of the fixed effect. The subgroup analysis was done based on the type of education (telephone, theory based, and group/individual), time of education (pregnancy/postpartum), participants (primiparous/multiparous), setting (hospital/health center), follow-up period, region, and study quality. Publication bias was assessed using the funnel plot, and Egger's and Begg's tests (Figure 3).

## 3. Results

The current study was updated until December 2020. A total of 1919 articles were extracted in the primary search based on the search strategy from the following databases: Scopus (129), ISI (515), PubMed (750), Mag-Iran (221), Cochrane (208), and other sources (96). Then, we excluded 640 duplicate articles, 1189 in the title and abstract review, and finally, 47 articles after the full-text review. Four papers had the inclusion criteria, we decided to exclude them from this analysis because one was a conference abstract, two papers had non-English language, and one paper due to lack of reporting the mean of self-efficacy, and we did not receive usable data from the author. A total of 40 articles with 5743 subjects that met the criteria for inclusion were retrieved in this systematic review. All extracted articles were published in Persian or English. The quality assessment of articles was performed by two reviewers, independently. A flowchart of the extracted articles and selection procedure is shown in Figure 1.

### 3.1. Characteristics of Included Studies

The characteristics of the 40 RCT studies included in the analysis are summarized in Figure 2. Of these, significant increases (*p* < 0.05) in BSE were reported in 26 of the included studies, which received breastfeeding education, and 14 studies reported no effect of education on BSE. Regarding the format of classes, 12 studies were based on group education that seven of them were based on theory. In 19 studies, educational tools such as booklets, pamphlets, video, and fillip cart were used for education. Moreover, women in 20 studies were educated with phone messages or telephone calling for follow-up. Based on the timing of education, the majority of interventions (31 studies) were done during pregnancy. The time of postpartum follow-up was varied at discharge to 24 weeks after childbirth. Regarding the region of the study, 18 studies were conducted in Iran and 2 studies were in Australia and Canada. One study was conducted in 9 countries including Croatia, Denmark, Hong Kong, Iraq, Japan, Spain, Thailand, and Turkey. The five and four study was conducted in the USA and Brazil, respectively. Regarding the study quality, one study had a score of 4, eight studies had score 5, twenty-one studies had score 6, four studies had score 7, and six studies had a score of 8. In all studies, self-efficacy was measured using Dennis breastfeeding self-efficacy (Table 1).

### 3.2. Main Results

**Figure 4:**
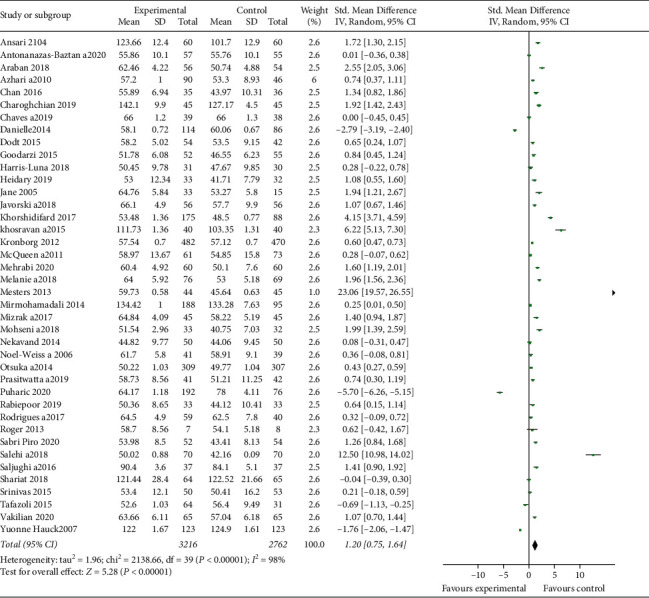
Forest plot of studies that investigated the influence of breastfeeding education on the self-efficacy of breastfeeding.

After quality assessment of studies, the results of 40 studies were included in the meta-analysis. The overall results demonstrated that educational intervention has a positive impact on BSE (pooled SMD = 1.20; 95% CI = 0.75–1.64, *p* value <0.001) (Figure 4). The high heterogeneity was seen among included studies (*I*^2^ = 98%, *p* < 0.001) (Figure 4). Egger's and Begg's tests were conducted to explore the publication bias in our meta-analysis (Figure 3). The funnel plot in Figure 3 shows symmetrical funnel plots among studies. There was no significant publication bias in this study based on Egger's and Begg's tests (*p*=0.790 and *p*=0.107, respectively).

### 3.3. Subgroup Analysis

The subgroup analyses were conducted based on the type of education (theory based, telephone, and group/individual), time of education (pregnancy/postpartum), participants (primiparous/multi), follow-up period, region, study quality, and setting (hospital/health centers). Our findings showed that the effect of theory-based education on BSE (SMD = 2.56; CI = 1.80–3.32) was more than that of non-theory-based education (SMD = 0.64; CI = 0.11–1.17), breastfeeding education during pregnancy (SMD = 1.76; 95% CI = 1.08–2.26) was more effective compared with the postpartum period (SMD = 0.08; 95% CI = -0.48–0.65), the use of phone for training or follow-up (SMD = 0.80; 95% CI = 0.04–1.56) was less effective compared with direct education (SMD = 1.57; 95% CI = 1–2.14), the effect of education on primiparous (SMD = 1.21; 95% CI = 0.57–1.86) was more than that of the multiparous (SMD = 1.09; 95% CI = 0.45–1.74), breastfeeding education in health centers (SMD = 2.31; 95% CI = 1.46–3.17) was more effective than in the hospital setting (SMD = 0.36; 95% CI = −0.15–0.87), the effect of education in Asia (SMD = 1.70; 95% CI = 1.21–2.20) was more than that in the other regions (SMD = 0.46; 95% CI = −0.30–1.22), the effect of education in higher-quality studies (SMD = 1.52; 95% CI = 1.01–2.04) was more than that in the lower-quality studies (SMD = 0.10; 95% CI = −0.61–0.81), the group education (SMD = 1.48; 95% CI = 0.61–2.35) was more effective on BSE than the individual education (SMD = 0.99; 95% CI = 0.46–1.52), but the heterogeneity of all subgroups was high. The subgroup analyses based on the follow-up period showed that education in the first week of postpartum had most effect on BSE (SMD = 1.49; 95% CI 1.36–1.61) than the others. However, the heterogeneity of studies in 6 weeks was least rate (*I*^2^ = 77.4%) (Table 2).

### 3.4. Sensitive Analysis

**Figure 5:**
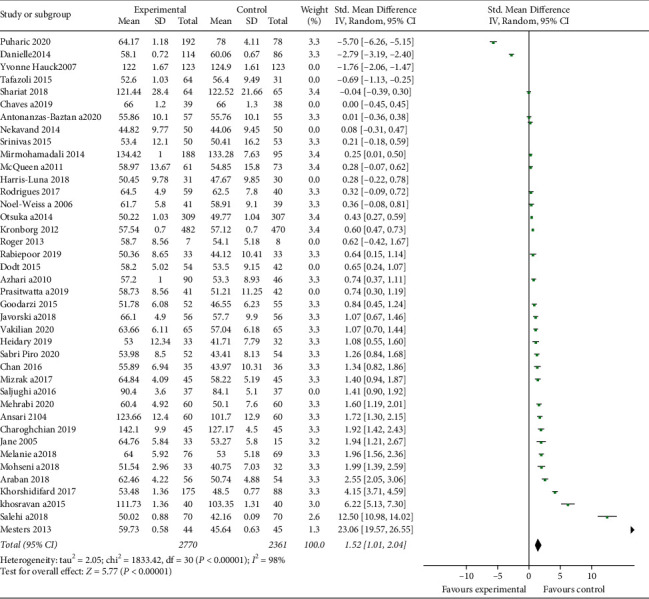
Sensitive analysis based on quality of studies.

For sensitive analysis, we excluded 9 studies that have low quality. The sensitive analysis showed that there were no obvious changes after excluding the studies (Figure 5). Therefore, our results were reliable.

## 4. Discussion

The results from the pooled RCT data highlight the positive impact of educational intervention on the self-efficacy of breastfeeding compared with the usual/standard care. However, substantial heterogeneity was high across the included studies. The subgroup analysis showed that the educational intervention was based on theory, group class format, direct method education, doing during pregnancy, on primiparous women, and health center setting, and the Asian region has a more effect on BSE than the others. The time of postpartum follow-up for evaluating the effect of educational intervention on BSE was a considerable point in the subgroup analysis. Accordingly, the effectiveness of education up to 6 weeks' follow-up period was significantly more than the other period with low heterogeneity among the included studies. Despite the high rate of heterogeneity, education in Asia was more effective than in other regions.

Galipeau et al. conducted a meta-analysis of 9 randomized control trials and quasi-experimental studies to evaluate the effectiveness of all types of prenatal interventions either educational, support, or psychosocial on breastfeeding self‐efficacy up to 4–6 weeks. The included studies were published from 2006 to 2016 in two English or French languages. They reported that overall interventions had a positive effect on breastfeeding self‐efficacy compared with usual care. However, the quality of the included study was low, and the heterogeneity was high. According to the subgroup analysis, they reported that interventions based on theory and direct education methods were more effective than the others. Overall, our result was consistent with the results of Galilean's study in the term of the type and method of education. However, the Galipeau study was not conducting subgroup analysis based on the type of study, and in other subgroups, the sample size was small [56].

Also, in 2019, Ghasemi et al. [8] conducted a systematic review of 21 both randomized control trials and quasi-experimental studies to evaluate the effectiveness of the theory-based intervention (educational and noneducational) on self-efficacy of Iranian women. The included articles were conducted on the Iranian population that was published in the Persian and English languages from 2010 to 2019. They were reported that the breastfeeding self-efficacy of mothers in the theory-based intervention group was more than the routine care group regardless of educational and noneducational intervention. Overall, our results were consistent with the results of Ghasemi's study in terms of theory-based intervention. However, Ghasemi's study was not doing meta-analysis and included studies were only in the Iranian population.

Also, Brockway et al. [13] in August 2016 performed a meta-analysis on 11 studies, both randomized control trials and quasi-experimental studies. Their study examined the effect of all interventions, whether education, support, counseling, or even screening and mechanical interventions on the BSE. In the event that these interventions are not of the same type, this issue has effect on their results. On the other hand, they reported high heterogeneity among included studies that indicated low quality of included studies. Then, a subgroup analysis showed that only education but not support has a positive impact on the BSE. In contrast to our results, they reported only intervention during the postpartum period but not prenatal intervention, improved BSE. It seems that the difference in the number of included studies in the meta-analysis and different inclusion criteria may contribute to this difference. Their study used only results of 3 studies during the postpartum and 2 studies during the prenatal period that were a combination of support and education. While in our study, 29 studies during prenatal and 11 studies during the postpartum period only with the educational intervention were included. However, we observe that education in healthcare centers was more effective than in the hospital settings, while in their results, education only in a combination setting (hospital and community) affected the BSE. Similar to their study, theory-based education was more effective at improving BSE.

We observe that theory-based education in comparison with non-theory-based education can more improve mother's BSE. Therefore, it is better to use theory-based educational intervention for educating these women. These results are consistent with the theory of self-efficacy, which states that modeling with practice is an effective way to increase self-efficacy [4]. Our results are in line with a previous meta-analysis, which is conducted by Chipojola et al. on 23 randomized controlled trial studies in 2019 [57]. This study indicated the overall effectiveness of educational programs based on theory (the theory of breastfeeding self-efficacy or theory of planned behavior) on the breastfeeding outcomes, and BSE. A high heterogeneity was reported among the included studies that indicated low-quality evidence. The subgroup analysis showed that mothers who received education based on the breastfeeding self-efficacy theory, providing in a hospital setting and developing countries, had a significantly higher score of BSE. Our finding is inconsistent with their results that indicate that education in healthcare centers is more effective than hospital setting. Also, in Guo et al.'s study, there was not seen any significant difference in the term of class format, time of education, and mode of class (face-to face or mobile education) on the BSE. It seems that the differences in inclusion criteria (only theory-based education) and number of included studies are caused by these differences. Another meta-analysis indicated a higher rate of exclusive breastfeeding among mothers who underwent interventions based on the theory of planned behavior [58]. Based on this theory, breastfeeding behaviors are influenced by attitudes, subjective norms, and perceived behavioral control [58]. During these sessions, mothers were taught the importance of breastfeeding and to create a positive attitude with invited influential people, as well as improved their perception of breastfeeding support [58].

One of the important findings of this study was that the effect of education on the BSE was significantly greater in Asian countries than in other countries. Therefore, it is important to pay attention to breastfeeding values in each country. Factors such as religion, tradition, culture, beliefs, and customs can affect breastfeeding self-efficacy.

Moreover, we observe that the most effective education on BSE was in the first week after discharge. This relationship was seen even up to the 24th week of childbirth. Therefore, it is important to continue education to increase women's BSE.

### 4.1. Strengths and Limitations

One main strength of the current systematic review was that a large number of electronic databases, as well as hand-search, were comprehensively explored to yield maximum relevant articles on this field. However, the quality assessment of methodology and data extraction were done by multiple reviewers. Also, the subgroup analysis was performed based on several factors that previous studies did not consider them. Furthermore, we excluded quasi-experimental studies, and analysis was done only on RCTs; therefore, the best quality of evidence was available.

Despite the study strengths, the results have inherent limitations. In the first place, significant heterogeneity was seen among the included studies. Even after sensitive analysis, the level of heterogeneity was high; therefore, the quality of evidence is low. Although we attempted to make the included studies be like regarding the methodology (only RCTs) and type of intervention (only education), it is not fully achieved even under ideal conditions. Furthermore, regarding the inherent of this study, publication bias is inevitable due to some nonsignificant results that might not have been published. However, included articles that were published only in English and Persian may limit the generalizability to other populations, and this might cause selection bias.

## 5. Conclusion

Breastfeeding education is considered an influential factor in the improvement of BSE. It is recommended that these interventions are better based on the theory, in healthcare setting, a group class format, during pregnancy, with direct method format, and continued to the first week of postpartum. Considering these issues in designing, an educational intervention provides an important opportunity for health professionals to increase mothers' confidence for breastfeeding when they encounter breastfeeding problems.

## Figures and Tables

**Table 1:** Summary of studies of the association between educational intervention and breastfeeding self-efficacy.

Author, year (location)	Study design	Groups (*n*)	Participants and setting	Time and format of classes	Intervention	Follow-up period	Tools and outcome	Results
Ali et al. Iran [18]	RCTs	Group 1: intervention, *n* = 95/100 Group 2: intervention, *n* = 93/100 Group 3: control, *n* = 95/100	Healthy primiparous women **Setting:** hospital	Postpartum (individual class) Telephone = no Theory = no	Group 1: one session, direct face-to-face educational program (20 min) Group 2: one session, indirect educational program using video CD + pamphlet (30 min) Group 3: routine care	Up to 3 months after childbirth	Breastfeeding self-efficacy tools: Dennis breastfeeding self-efficacy (33 items)	Three months after childbirth, a significant difference was observed between the three groups in the mean of breastfeeding self-efficacy (*p* < 0.001). But there was not a significant difference between each intervention groups compared to control group (*p* > 0.05).

Heydari et al. Iran [19]	RCTs	Group 1: intervention, *n* = 33/35 Group 2: control, *n* = 32/35	Healthy primiparous women **Setting:** childbirth preparation classes	Pregnancy (group class) Telephone = yes Theory = no	Group1: one session, direct face-to-face educational program (45–60 min) + **telegram** chat up to 4 months after child birth Group 2: routine care	Up to four months after childbirth	Breastfeeding self-efficacy tools: Dennis breastfeeding self-efficacy (14 items)	Four months after childbirth, a significant difference was observed between the two groups in the mean of breastfeeding self-efficacy (*p* < 0.001).

Azhari et al. Iran [20]	RCTs	Group 1: intervention, *n* = 45 Group 2: intervention, *n* = 45 Group 3: control, *n* = 46	Healthy primiparous women **Setting:** hospital	Postpartum (individual class) Telephone = no Theory = no	Group 1: one session direct face-to-face educational program (20–40 min) Group 2: one session indirect educational program using the images Group 3: routine care	1, 4, and 8 weeks after childbirth	Breastfeeding self-efficacy tools: self-efficacy 14 items	After three-stage follow-up, a significant difference was observed between the three groups in the mean of breastfeeding self-efficacy (*p* < 0.001). The highest mean was for the indirect educational group

Godarzi et al. Iran [21]	RCTs	Group 1: intervention, *n* = 52 Group 2: control, *n* = 55	Healthy primiparous women **Setting:** public health center	Pregnancy (group class) Telephone = no Theory = no	Group 1: two sessions (one session in third trimester and one session after child birth. Peer education method was held using lecture and group discussion approach Group 2: routine care	Up to 8 weeks after child birth	Breastfeeding self-efficacy tools: Dennis breastfeeding self-efficacy (14 items)	Eight weeks after childbirth, a significant difference was observed between the two groups in the mean of breastfeeding self-efficacy (*p* < 0.001).
Chan et al. Hong Kong [22]	RCTs	Group 1: intervention, *n* = 35/35 Group 2: control, *n* = 36/36	Healthy primiparous women **Setting:** hospital	Pregnancy (group class) Telephone = yes Theory = yes	Group1: one session a self-efficacy-based breastfeeding, educational program was held as a workshop + two telephone call after childbirth (30–60 mn) Group 2: routine care	Up to 2 weeks after child birth	Breastfeeding self-efficacy tools: Dennis breastfeeding self-efficacy (14 items)	Two weeks after childbirth, a significant difference was observed between the two groups in the mean of breastfeeding self-efficacy (*p* < 0.01).

Ansari et al. Iran [23]	RCTs	Group 1: intervention, *n* = 60/65 Group 2: control, 60/65	Healthy pregnancy **Setting:** public health center	Pregnancy (individual class) Telephone = yes Theory = yes	Group 1: two-session self-efficacy-based educational program with two-day interval for two hours + phone calls Group 2: routine care	One month after child birth	Breastfeeding self-efficacy tools: Dennis breastfeeding self-efficacy (33 items)	One month after childbirth was a significant diﬀerence between two groups regarding self-efficacy mean (*p* < 0.001).

Antoñanzas-Baztán et al. Spain [11]	RCTs	Group 1: intervention *n* = 57/59 Group 2: control, *n* = 55/59	Healthy pregnancy **Setting:** hospitals and community center	Pregnancy (group class) Telephone = yes Theory = no	Group 1: three-session educational program was held. In 28–39 gestational weeks, before discharge, and phone call 48–72 hours after childbirth Group 2: usual care	4 and 8 weeks and 6 months after child birth	Breastfeeding self-efficacy tools: Dennis breastfeeding self-efficacy (14 items)	After three-stage follow-up, there was **no** significant difference between the two groups on BSES (*p* > 0.05).

Piro and Ahmed Iraq [24]	RCTs	Group 1: intervention, *n* = 52/65 Group 2: control, *n* = 54/65	Healthy pregnancy **Setting:** public health center	Pregnancy (group class) Telephone = no Theory = yes	Group 1: two-session self-efficacy-based educational program was held with two-day interval, each session lasting for 60–90 min + booklet + video Group 2: routine care	Up to two months after child birth	Breastfeeding self-efficacy tools: Dennis breastfeeding self-efficacy (14 items)	Two months after childbirth, there was a significant diﬀerence between two groups regarding self-efficacy mean (*p* < 0.001).

Mohseni et al. Iran [25]	RCTs	Group 1: intervention, *n* = 33/35 Group 2: control, *n* = 32/35	Healthy primiparous women **Setting:** public clinics, home visit	Pregnancy (individual class) Telephone = yes Theory = no	Group 1: 3-session educational program was held per week in their **house** + an educational pamphlet + one visit home after childbirth Group 2: routine care	1,2, and 6 weeks after childbirth	Breastfeeding self-efficacy tools: Dennis breastfeeding self-efficacy (14 items)	After three-stage follow-up, there was a significant difference between the two groups (*p* < 0.001).

Rabiepoor et al. Iran [26]	RCTs	Group 1: intervention, *n* = 33 Group 2: control, *n* = 33	Healthy pregnancy **Setting:** Public health center	Pregnancy (individual class) Telephone = yes Theory = no	Group 1: two-session couple educational program with 4-week interval + **telephone** call over study time. Training package included prenatal and postnatal care and lactation Group 2: routine care	Up to one month after child birth	Breastfeeding self-efficacy tools: Dennis breastfeeding self-efficacy (14 items)	One month after childbirth, there was a significant diﬀerence between two groups regarding self-efficacy mean (*p* < 0.017).
Puharić et al. Croatia [27]	RCTs	Group 1: intervention, *n* = 129/136 Group 2: intervention, *n* = 103/128 Group 3: control group, *n* = 123/136	Healthy primiparous women **Setting:** hospitals	Pregnancy (individual class) Telephone = yes Theory = no	Group 1: three-session educational program included breastfeeding and parenting booklet + four **proactive telephone** calls (one session in pregnancy and three after delivery, at 2, 6, and 10 weeks) Group 2: one-session educational program included pregnancy booklet + four proactive **telephone** calls (one in pregnancy and three after delivery, at 2, 6, and 10 weeks) Group 3: routine care	Up to 3 months	Breastfeeding self-efficacy tools: Dennis breastfeeding self-efficacy (14 items)	Three months after childbirth, there was a significant diﬀerence between three groups regarding self-efficacy mean (*p* < 0.001).

Charoghchian Khorasani et al. Iran [28]	RCTs	Group 1: intervention, *n* = 45 Group 2: control, *n* = 45	Healthy primiparous women **Setting:** public health center	Pregnancy (group class) Telephone = no Theory = yes	Group 1: one-session **self-efficacy**-based educational program was held using lectures, role playing, posters, and CD + health literacy Group 2: routine care	Up to 3 months	Breastfeeding self-efficacy tools: Dennis breastfeeding self-efficacy (33 items)	Three months after childbirth, a significant difference was observed between the two groups in the mean of breastfeeding self-efficacy (*p* < 0.001).

Vakilian et al. Iran [29]	RCTs	Group 1: intervention, *n* = 65 Group 2: control, *n* = 65	Healthy primiparous women **Setting:** hospital	Postpartum (individual class) Telephone = no Theory = no	Group 1: **home-based** educational program using pamphlet + CD Group 2: routine care	Up to 4 weeks	Breastfeeding self-efficacy tools: Dennis breastfeeding self-efficacy (14 items)	Four weeks after childbirth, a significant difference was observed between the two groups in the mean of breastfeeding self-efficacy (*p* < 0.001).

Shariat et al. Iran [12]	RCTs	Group 1: intervention, *n* = 64 Group 2: control, *n* = 65	Healthy primiparous women **Setting:** hospital	Postpartum (individual class) Telephone = yes Theory = no	Group 1: one-session educational program using pamphlet + CD Group 2: routine care	Up to 6 months	Breastfeeding self-efficacy tools: Dennis breastfeeding self-efficacy (33 items)	After six months, there was **no** significant difference between the two groups on BSES (*p* > 0.05).

Rodrigues et al. Brazil [30]	RCTs	Group 1: intervention, *n* = 59/104 Group 2: control, *n* = 40/104	Healthy pregnancy **Setting:** hospital	Postpartum period (group class) Telephone = no Theory = no	Group 1: one-session educational program using flip chart (40 min) Group 2: routine care	15, 30, 60, 90, and 120 days after child birth	Breastfeeding self-efficacy tools: Dennis breastfeeding self-efficacy (14 items)	After five-stage follow-up, there was a significant difference between the two groups (*p* < 0.001).
Chaves et al. Brazil [31]	RCTs	Group 1: intervention, *n* = 39/66 Group 2: control, *n* = 38/66	Healthy pregnancy **Setting:** hospital	Postpartum period (individual class) Telephone = yes Theory = no	Group 1: one-session face-to-face educational program + three **telephone** calls in 7, 15, and 30 days after discharge (70 min) Group 2: routine care	2 and 4 months after child birth	Breastfeeding self-efficacy tools: Dennis breastfeeding self-efficacy (14 items)	After two months, there was **no** significant difference between the two groups on BSES (*p* > 0.05). After four months, there was a significant difference between the groups (*p* < 0.01).

Dodt et al. Brazil [32]	RCTs	Group 1: intervention, *n* = 54/101 Group 2: control, *n* = 42/100	Healthy pregnancy **Setting:** hospital	Postpartum period (individual class) Telephone = yes Theory = no	Group 1: three-session educational program using flip chart in 6 hours postpartum, before discharge, and 2 months after child birth by telephone contact Group 2: routine care	Up to 2 months	Breastfeeding self-efficacy tools: Dennis breastfeeding self-efficacy (14 items)	After two months, there was a significant difference between the two groups (*p* < 0.03).

Srinivas et al. USA [33]	RCTs	Group 1: intervention, *n* = 50/63 Group 2: control, *n* = 53/63	Healthy pregnancy **Setting:** hospital	Pregnancy (individual class) Telephone = yes Theory = no	Group 1: 11-session peer educational program was held, one session 28^th^ weeks of pregnancy, one session within 3 to 5 days after delivery, weekly to 1 month, every 2 weeks up to 3 months, and once at 4 months using telephone call Group 2: routine care	Up to 6 weeks after child birth	Breastfeeding self-efficacy tools: Dennis breastfeeding self-efficacy (14 items)	After six weeks, there was **no** significant difference between the two groups on BSES (*p* > 0.05).

Nekavand et al. Iran [34]	RCTs	Group 1: intervention, *n* = 50 Group 2: control, *n* = 50	Healthy primiparous women **Setting:** Hospital	Postpartum period (individual class) Telephone = no Theory = no	Group 1: one-session educational program within 5 hours after child birth + booklet Group 2: routine care	Up to one month	Breastfeeding self-efficacy tools: Dennis breastfeeding self-efficacy (14 items)	One month after childbirth, there was **no** significant difference between the two groups on BSES (*p* > 0.05).

Kronborg et al. Denmark [35]	RCTs	Group 1: intervention, *n* = 582/603 Group 2: control, 575/590	Healthy primiparous women **Setting:** hospital	Pregnancy (couple group class) Telephone = no Theory = no	Group 1: one-session educational program was held + lectures and discussions + shown a film for 9 hours Group 2: routine care	Up to six weeks after child birth	Breastfeeding self-efficacy tools: Dennis breastfeeding self-efficacy (14 items)	After six weeks, there was **no** significant difference between the two groups on BSES (*p* > 0.05).

Javorski et al. Brazil [36]	RCTs	Group 1: intervention, *n* = 56/66 Group 2: control, 56/66	Healthy pregnancy **Setting:** basic health units	Pregnancy (individual class) Telephone = no Theory = no	Group 1: educational program was held using flip chart included picture and text Group 2: routine care	2, 4, and 8 weeks after child birth	Breastfeeding self-efficacy tools: Dennis breastfeeding self-efficacy (14 items)	After 2, 4, and 8 weeks, a significant difference was observed between the two groups in the mean of breastfeeding self-efficacy (*p* < 0.001).
Araban et al. Iran [10]	RCTs	Group 1: intervention 56/60 Group 2: control, *n* = 54/60	Healthy primiparous women **Setting:** prenatal clinics	Pregnancy (group class) Telephone = no Theory = yes	Group 1: two-session group-based educational program was held + booklet and images + sending biweekly text messages up to 8 weeks after childbirth Group 2: routine care	Up to 8 weeks	Breastfeeding self-efficacy tools: Dennis breastfeeding self-efficacy (14 items)	After 8 weeks, a significant difference was observed between the two groups in the mean of breastfeeding self-efficacy (*p* < 0.001).

Harris-Luna and Badr. California [37]	RCTs	Group 1: intervention, *n* = 31 Group 2: control, *n* = 30	Healthy pregnancy **Setting:** obstetric clinic	Pregnancy (individual class) Telephone = yes Theory = no	Group 1: bilingual Spanish-English educational program was held in three sessions (2 hours) + **telephone** support weekly for the first 4 weeks and then biweekly for up to 12 weeks after child birth Group 2: routine care	Up to 12 weeks after child birth	Breastfeeding self-efficacy tools: Dennis breastfeeding self-efficacy (14 items)	After 12 weeks, a significant difference was observed between the two groups in the mean of breastfeeding self-efficacy (*p* < 0.001).

Mizrak et al. Turkey [38]	RCTs	Group 1: intervention, *n* = 45 Group 2: control, *n* = 45	Healthy primiparous women **Setting:** health centers	Pregnancy (group class) Telephone = yes Theory = yes	Group 1: two educational programs were held in a week using video (90–80 min) + home visit in 1, 4, and 8 weeks or telephone call Group 2: standard care	1, 4, and 8 weeks after child birth	Breastfeeding self-efficacy tools: Dennis breastfeeding self-efficacy (14 items)	After 1, 4, and 8 weeks, a significant difference was observed between the two groups in the mean of breastfeeding self-efficacy (*p* < 0.001).

McQueen et al. Toronto [39]	RCTs	Group 1: intervention, *n* = 61/68 Group 2: control, *n* = 73/81	Healthy primiparous women **Setting:** hospital	Postpartum period (individual class) Telephone = yes Theory = yes	Group 1: two-session educational program based on self-efficacy theory was conducted in hospital, and one session was conducted by telephone within 1 week of discharge from hospital Group 2: standard care	4 and 8 weeks after child birth	Breastfeeding self-efficacy tools: Dennis breastfeeding self-efficacy (14 items)	After 4 and 8 weeks, there was **no** significant difference between the two groups on BSES (*p* > 0.05).

Prasitwattanaseree et al. Thailand [40]	RCTs	Group 1: intervention, *n* = 41/48 Group 2: control, *n* = 42/49	Healthy primiparous women **Setting:** hospital	Pregnancy (individual class) Telephone = yes Theory = no	Group 1: twelve educational programs were held (2 sessions during pregnancy per week + 1, 2, and 3 days after birth + 7 days, 1 month, 6 weeks, and 3 and 6 months at home with telephone call Group 2: usual care	At discharge and 6 weeks after child birth	Breastfeeding self-efficacy tools: Dennis breastfeeding self-efficacy (14 items)	At discharge, there was **no** significant difference between the two groups on BSES (*p* > 0.05). After 6 weeks, a significant difference was observed between the two groups in the mean of breastfeeding self-efficacy (*p* < 0.001).
Moudi et al. Iran [41]	RCTs	Group 1: Intervention, *n* = 32/36 Group 2: intervention, *n* = 32/36 Group 3: control, *n* = 31/32	Healthy primiparous women **Setting:** health centers	Pregnancy (individual class) Telephone = yes Theory = no	Group 1: peer support training was held in four sessions. One session was face to face in 36–38 weeks, and three sessions were done using telephone call in 1, 2, and 3 weeks after child birth Group 2: provider training was held in four sessions. Two sessions were face to face, and the next two sessions were held in the 1 and 3 weeks after child birth by telephone call Group 3: standard care	8 weeks after child birth	Breastfeeding self-efficacy tools: Dennis breastfeeding self-efficacy (14 items)	After 8 weeks, there was **no** significant difference between the three groups on BSES (*p* > 0.05). BSES at the end of the eighth week was significantly increased in peer support compared to provider groups (*p* < 0.05).

Saljughi et al. Iran [42]	RCTs	Group 1: intervention, *n* = 37 Group 2: control, *n* = 37	Healthy pregnancy **Setting:** health centers	Pregnancy group class) Telephone = no Theory = no	Group 1: one-session educational program using role playing approach (90 min) Group 2: routine care	One week and 1 month after child birth	Breastfeeding self-efficacy tools: breastfeeding self-efficacy (14 items)	After 1 week and 1 month, a significant difference was observed between the two groups in the mean of breastfeeding self-efficacy (*p* < 0.001).

Khorshidifard et al. Iran [43]	RCTs	Group 1: intervention, *n* = 88 Group 2: intervention, *n* = 88 Group 3: control, *n* = 88	Healthy primiparous women **Setting:** health centers	Pregnancy individually + small group class) Telephone = no Theory = no	Group 1: direct face-to face-individually educational program in three sessions using lecture, discussion, and role playing approach Group 2: small group educational program was held in three sessions Group 3: routine care	After last session of education and after child birth	Breastfeeding self-efficacy tools: Dennis breastfeeding self-efficacy (14 items)	After last session of education and after child birth, there was a significant difference between three groups in the mean of breastfeeding self-efficacy (*p* < 0.001).

Otsuka et al. Japan [44]	RCTs	Group 1: intervention, *n* = 136 Group 2: intervention *n* = 239 Group 3: control, *n* = 140 Group 4: control, *n* = 266	Healthy pregnancy **Setting:** baby-friendly hospitals (BFHs)	Pregnancy (individual class) Telephone = no Theory = yes	Groups 1 and 2: self-efficacy workbook in six sections was completed by women Groups 3 and 4: routine care	At discharge and 4 weeks after child birth	Breastfeeding self-efficacy tools: Dennis breastfeeding self-efficacy (14 items)	After controlling for potential confounding factors and time, the intervention resulted in an increase in the BSES-SF total score through 4 weeks postpartum in BFHs (*p* < 0.001), but it had no effect on breastfeeding self-efficacy *p* < 0.05).
Khosravan^,^ et al. Iran [45]	RCTs	Group 1: intervention *n* = 40 Group 2: control *n* = 40	Healthy primiparous women **Setting:** hospital	Pregnancy (individual class) Telephone = no Theory = yes	Group 1: six-session educational program using problem solving Group 2: routine care	After last session of education and 3 months after child birth	Tools: Dennis breastfeeding self-efficacy (33 items)	After last session of education and after child birth, there was a significant difference between two groups in the mean of breastfeeding self-efficacy (*p* < 0.001).

Salehi et al. Iran [46]	RCTs	Group 1: intervention *n* = 70 Group 2: intervention *n* = 70 Group 3: control *n* = 70	Healthy primiparous women **Setting:** health center	Pregnancy (group class) Telephone = yes Theory = no	Group 1: a **motivational** interview was held preweek in five sessions + three sessions of **telephone** counseling in 3–5 days, 1 and 4 months after discharge + **telegram** chat Group 2: one session of lecture (2 hours) + question and answer panel Group 3: routine care	2, 4, and 6 months after child birth	Tools: Dennis breastfeeding self-efficacy (14 items)	In the lecture and control group, there was a significant increase until the second month (*p* < 0.001), and self-efficacy decreased in months 4 and 6 compared to the second months (*p* > 0.05).

Lutenbacher et al. USA [47]	RCT	Group 1: intervention *n* = 76 Group 2: control *n* = 69	Participant healthy pregnancy **Setting:** health center	Pregnancy individual class Telephone = yes Theory = no	Group 1: educational program based on the maternal infant health outreach worker (MIHOW) model by peer mentors (40 h) Group 2: routine care (a minimal education intervention MEI)	2 weeks and 2 and 6 months after childbirth	Tools: Dennis breastfeeding self-efficacy (14 items)	After three-stage follow-up, there was a significant difference between the two groups (*p* < 0.001).

Mesters et al. Netherland [48]	RCTs	Group 1: intervention, *n* = 44 Group 2: control, *n* = 45	Healthy pregnancy **Setting:** health center	Pregnancy (individual class) Telephone = no Theory = yes	Group 1: four-session educational program using a **theory-based** booklet and pre- and postnatal home visits (2 sessions of face to face and one session as a home visit in prenatal + 1 session home visit in postnatal) Group 2: routine care	3 months after child birth	Tools: Dennis breastfeeding self-efficacy (14 items)	A statistically significant difference was observed between 8 months of pregnancy and 3 months postpartum in which self-efficacy expectation increased in both groups (*p* < 0.05).

Edwards et al. USA [49]	RCTs	Group 1: intervention, *n* = 7 Group 2: control, *n* = 8	Healthy primiparous women **Setting:** hospital	Pregnancy individual Telephone = no Theory = no	Group 1: educational program using a computer agent Group 2: usual care	At discharge	Tools: Dennis breastfeeding self-efficacy (14 items)	At discharge, **no** significant difference was observed between the three groups on BSES (*p* > 0.05).
Noel-Weiss et al. Canada [50]	RCTs	Group 1: intervention, *n* = 41 Group 2: control, *n* = 39	Healthy primiparous women **Setting:** hospital	Postpartum group class Telephone = yes Theory = yes	Group 1: the intervention was a 2.5-hour prenatal breastfeeding workshop designed using Bandura's theory of self-efficacy and adult learning principles Group 2: routine care	4 and 8 weeks after childbirth	Tools: Dennis breastfeeding self-efficacy (14 items)	After 4 weeks, there was a significant difference between the two groups (*p*=0.02). After 8 weeks, **no** significant difference was observed between the two groups (*p* > 0.05).

Hauck et al. Australia [51]	RCTs	Group 1: intervention, *n* = 123 Group 2: control, *n* = 123	Healthy primiparous women **Setting:** hospital	Pregnancy individual class Telephone = no Theory = no	Group 1: the intervention was a 3-hour prenatal breastfeeding workshop + breastfeeding journal Bandura's theory Group 2: routine care	12 weeks after child birth	Tools: Dennis breastfeeding self-efficacy (33 items)	After 12 weeks, **no** significant difference was observed between the two groups (*p* > 0.05).

Mehrabi et al. Iran [52]	RCTs	Group 1: intervention, *n* = 60 Group 2: control, *n* = 60	Healthy women **Setting:** health center	Pregnancy individual class Telephone = yes Theory = no	Group 1: mobile messaging on breastfeeding self-efficacy educational program Group 2: routine care	After 2 weeks after child birth	Tools: Dennis breastfeeding self-efficacy (14 items)	After 2 weeks, there was a significant difference between the two groups(*p*=0.001).

Schlickau, Nebraska, USA [53]	RCTs	Group 1: intervention, *n* = 33 Group 2: control, *n* = 15	Healthy primiparous women **Setting:** health	Pregnancy individual	Group 1: one-session educational program based on **Pender's health promotion model (HPM)** and support after child birth during the follow-up period Group 2: standard care	2 weeks after child birth	Tools: Dennis breastfeeding self-efficacy (14 items) 2 weeks	After 2 weeks, there was a significant difference between the two groups(*p*=0.001).

Gallegos et al. Australia [54]	RCTs	Group 1: intervention, *n* = 114 Group 2: control, *n* = 86	Healthy women **Setting:** health center	Postpartum group class Telephone = yes Theory = no	Group 1: weekly educational massage for eight weeks + Facebook page involvement Group 2: standard care	8 weeks after child birth	Tools: Dennis breastfeeding self-efficacy (14 items)	After 8 weeks, **no** significant difference was observed between the two groups (*p* > 0.05).

Abuidhail et.al. Jordan [55]	A prospective RCT	Group 1: intervention, *n* = 56 Group 2: control, *n* = 56	**Participant:** healthy pregnancy	Pregnancy (individual class)	Group 1: two-session educational program was held using videos and images. It can be accessed by **website** with connecting Internet by computers or smart phones or any device + one notification text massage with mobile 3 days after last educational program Group 2: routine care	Up two weeks after child birth	Tools: Dennis breastfeeding self-efficacy (14 items)	After adjusting for preintervention scores, there was **no** significant difference between the experimental and control groups on postintervention scores on BSES (*p*=0.22).

**Table 2:** The result of subgroup analysis on the breastfeeding self-efficacy.

Subgroups	SMD (95% CI)	No. of study	*I* ^2^
Primiparous	1.21 (0.57–1.86)	24	98.5
Multiparous	1.09 (0.45–1.74)	16	97.6

Group	1.48 (0.61–2.35)	15	98.8
Individual	0.99 (0.46–1.52)	25	97.7

Phone	0.80 (0.04–1.56)	21	98.3
Direct education	1.57 (1.0–2.14)	19	98.1

Theory	2.56 (1.80–3.32)	11	96.9
No theory	0.64 (0.11–1.17)	29	98.3

Postpartum	0.08 (−0.48–0.65)	11	96.2
Pregnancy	1.67 (1.08–2.26)	29	98.5

Hospital	0.36 (−0.15–0.87)	21	98.0
Health center	2.31 (1.46–3.17)	19	98.2

Asia	1.70 (1.21–2.20)	23	97.1
Others	0.46 (−0.30–1.22)	17	98.6

Higher-quality studies	0.10 (−0.61–0.81)	9	95.6
Lower-quality studies	1.52 (1.01–2.04)	31	98.0

At discharge up to 1 week	1.49 (1.36–1.61)	9	96.9
2 weeks	1.18 (1.01–1.35)	7	85.6
4 weeks	0.64 (0.54–0.73)	13	86.5
6 weeks	0.61 (0.50–0.73)	4	77.4
8 weeks	0.53 (0.42–0.63)	16	97.8
12 weeks	−0.35 (−0.50–0.21)	8	99.2
16 weeks	0.87 (0.62–1.13)	4	98.8
24 weeks	0.93 (0.72–1.14)	4	98.9

## Data Availability

The data used to support the findings of this study are available upon request.
